# Structure and dissolution of silicophosphate glass

**DOI:** 10.1039/d2ra06707b

**Published:** 2022-12-07

**Authors:** Kazuya Takada, Tomoyuki Tamura, Toshihiro Kasuga

**Affiliations:** Division of Advanced Ceramics, Nagoya Institute of Technology Gokiso-cho, Showa-ku Nagoya 466-8555 Japan kasuga.toshihiro@nitech.ac.jp; Division of Applied Physics, Nagoya Institute of Technology Gokiso-cho, Showa-ku Nagoya 466-8555 Japan tamura.tomoyuki@nitech.ac.jp

## Abstract

P_2_O_5_–SiO_2_–Na_2_O–CaO glasses are promising therapeutic ion-releasing materials. Herein, we investigated the state of silicon (Si) in P_2_O_5_–SiO_2_–Na_2_O–CaO glass using a model with a composition of 55.0P_2_O_5_–21.3SiO_2_–23.7Na_2_O (mol%), incorporating a six-fold-coordinated silicon structure (^[6]^Si). The model was constructed using a classical molecular dynamics method and relaxed using the first-principles method. Further, we experimentally prepared glasses, substituting Na_2_O for CaO, to investigate the dissolution of glass with varying ^[6]^Si and PO_4_ tetrahedra (*Q*_P_^*n*^) distributions (*n* = number of bridging oxygens (BOs) to neighboring tetrahedra). ^[6]^Si in the glass model preferentially coordinated with *Q*_P_^3^. When Si was surrounded by phosphate groups, phosphorus (P) induced the formation of ^[6]^Si by elongating the Si–O distance, and ^[6]^Si acted like a glass network former (NWF). Na^+^ coordinated with ^[6]^Si–O–P bonds *via* electrostatic interactions with BO. ^31^P and ^29^Si magic-angle-spinning-nuclear-magnetic-resonance spectra of three experimental glass samples with the compositions of 55.0P_2_O_5_–21.3SiO_2_–*x*CaO–(23.7 − *x*)Na_2_O (mol%, *x* = 0, 12.4, and 23.7) showed that *Q*_P_^3^ and ^[6]^Si increased with increasing Na_2_O. When each glass powder was immersed in a tris-HCl buffer solution at 37 °C, the dissolution of NWF ions and network modifier (NWM) ions increased almost monotonically with time for all samples, indicating that the solubility of the samples was suppressed by the coexistence of CaO and Na_2_O, attributed to the delocalization of the electron distribution of P in the ^[6]^Si-coordinated *Q*_P_^3^ units compared to that in the P- or ^[4]^Si-coordinated *Q*_P_^3^ units, which reduces hydrolysis.

## Abstract 2

The solubility of P_2_O_5_–SiO_2_–Na_2_O–CaO glasses was suppressed by the coexistence of CaO and Na_2_O, attributed to the delocalization of the electron distribution of P in *Q*_P_^3^ units coordinated to the six-fold-coordinated Si.

## Graphical abstract

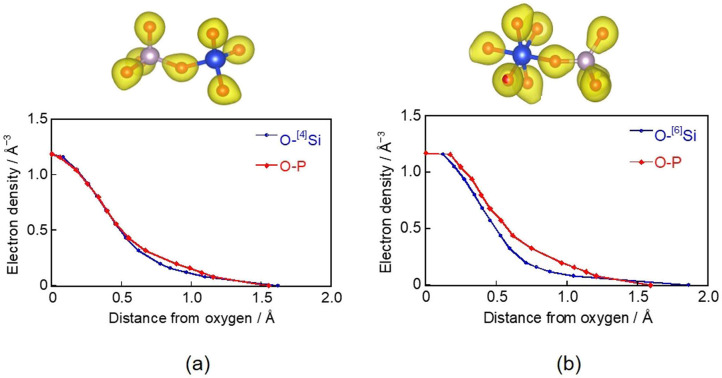

## 1 Introduction

Water-soluble phosphate glasses can promote bone regeneration by releasing inorganic ions as bone-formation-promoting factors. Calcium, phosphate, and silicate ions are involved in bone formation and promote the proliferation, differentiation, and mineralization of osteoblast-like cells at appropriate concentrations. The dissolution of these ions from the glass must be reasonably controlled.

The diffusion of glass network modifier (NWM) ions, such as Na^+^ and Ca^2+^ ions, in phosphate glass determines the solubility of the glass.^1^ In the dissolution of phosphate glasses, ion-exchange reactions (hydration reactions) between NWM ions and protons dominate, and NWM ions diffuse to the surface.^2–5^ To understand the relationship between the phosphate tetrahedral morphology (*Q*_P_^*n*^ unit: *n* is the number of bridging oxygen (BO)) and ion diffusion, we employed *ab initio* molecular dynamics (MD) simulations to study the dynamics of Na^+^ ions and protons in phosphate glass.^6^ As an example of a phosphate glass containing both *Q*_P_^2^ and *Q*_P_^3^ units, 55.0P_2_O_5_–21.3SiO_2_–23.7Na_2_O (mol%) glass was simulated using a model in which Na^+^ ions are replaced by protons, assuming a progressing state of its dissolution. When a proton was adsorbed on the nonbridging oxygen (NBO) of the *Q*_P_^3^ unit, it desorbed in a short time within 10 fs and readsorbed on the NBO on which protons had been adsorbed. On the other hand, when a proton was adsorbed on the NBO of the *Q*_P_^2^ unit, another proton coordinated before the adsorption could desorb sequentially, resulting in proton diffusion. When Na^+^ ions were present in the vicinity, proton adsorption on the *Q*_P_^2^ unit reduced the electrostatic interaction between Na^+^ and O^2−^ ions and induced the detachment of Na^+^ ions. This result explains the much early stages of the glass reaction with water. The solubility of the phosphate glass could be engineered by tuning their *Q*_P_^*n*^ distribution.

Dupree *et al.* reported that a six-fold-coordinated silicon (Si) structure (^[6]^Si) is readily formed in silicate glasses containing more than 40 mol% P_2_O_5_.^7^ In our previous study, we found that in P_2_O_5_–SiO_2_–Na_2_O–CaO glasses with high P_2_O_5_ content, ion dissolution is improved in an ultraphosphate glass with high ^[6]^Si content.^8^ We reported the ions release behavior for two types of ^[6]^Si-containing glasses with the P_2_O_5_ content of 45 and 50 mol%, fixed SiO_2_/Na_2_O/CaO ratios. The glass with high P_2_O_5_ content controlled the ion release amounts effectively. Note that the amounts of ions release decreased in silicophosphate glasses containing ^[6]^Si, whereas usually the amount of ions dissolved from SiO_2_-free P_2_O_5_–Na_2_O–CaO glasses increases with increasing P_2_O_5_ content. We predicted that the hydrolysis might be controlled by the formation of Si–O–P bonds between ^[6]^Si and *Q*_P_^3^. These results and prediction motivate us to investigate the relation between glass structure and solubility on the basis of the importance of the ^[6]^Si and *Q*_P_^*n*^ distribution. By clarifying this, it may be possible to design phosphate glasses so that their solubility can be controlled freely.

In this study, first we attempted visualizing the electron density distribution around ^[6]^Si–*Q*_P_^3^ using our earlier 55.0P_2_O_5_–21.3SiO_2_–23.7Na_2_O glass model and discussed the possible effect of this structure on its solubility. Then, we experimentally verified whether the solubility of P_2_O_5_–SiO_2_–(Na_2_O, CaO) glasses can be improved by varying ^[6]^Si and *Q*_P_^*n*^ distributions. It has been reported that the amount of ^[6]^Si formation in phosphate glass varies not only with P_2_O_5_ content but also with the types of NWMs (alkali and/or alkaline earth ions).^7,9^ In this study, we tried to form some ^[6]^Si and *Q*_P_^*n*^ distribution following this phenomenon to investigate the effect of the glass structure on the durability; 55.0P_2_O_5_–21.3SiO_2_–*x*CaO–(23.7 − *x*)Na_2_O glasses (*x* = 0, 12.4, and 23.1) with fixed P_2_O_5_ and SiO_2_ contents were primarily focused upon.

P_2_O_5_–Na_2_O–CaO glass has been widely studied, especially its structure and chemical durability.^1,10–13^ However, only a few attempts have been made to modulate its solubility by introducing ^[6]^Si. Silicon plays an effective role in bone formation. In this study, we studied the surroundings of ^[6]^Si in P_2_O_5_–SiO_2_–Na_2_O glass MD simulations and experimentally examined the solubility of glasses with different ^[6]^Si and *Q*_P_^*n*^ distributions by varying the amounts of Na_2_O and CaO.

## 2 Experimental section

### 2.1 Modeling of P_2_O_5_–SiO_2_–Na_2_O glass by classical MD simulation

In our previous report, we developed a 55.0P_2_O_5_–21.3SiO_2_–23.7Na_2_O (mol%) glass model using a classical MD program (DL_POLY).^14^ Details of the simulation can be found in our previous report.^6^ A system consisting of 510 atoms was melted at 1900 K for 100 ps and then cooled rapidly to 300 K at a rate of 2.0 K ps^−1^. An NVT ensemble with a Nosé–Hoover thermostat^15^ was used. The bond angles of O–Si–O and O–P–O bonds were controlled using a three-body screened harmonic potential.^16,17^ To reproduce a four-coordinated Si structure (^[4]^Si) and ^[6]^Si, the potential of *θ*_0_ = 109.47° and *k*_b_ = 250 eV rad^−1^ was assigned to the number of Si atoms corresponding to ^[4]^Si with reference to the coordination number distribution revealed by ^29^Si magic-angle-spinning-nuclear-magnetic-resonance (MAS-NMR) spectroscopy. The classical MD model was relaxed by density functional theory (DFT) calculations.^18,19^ We employed the projector augmented-wave (PAW) method^20,21^ with the generalized gradient approximation (GGA) with the Perdew–Burke–Ernzerhof functional^22^ for the exchange–correlation energy functional, as implemented in the Viena Ab initio Simulation Package code (VASP).^21,23^ We used only a single *k*-point (Gamma point) and plane waves with energies of up to 400 eV. Full structural optimization was performed using a conjugate-gradient method^24^ until the forces became smaller than 10 meV Å^−1^. No large structural changes, such as bond recombination, were observed. For the electronic structure analysis, we used a model consisting of 198 atoms prepared under similar conditions.

### 2.2 Glass sample preparation

55.0P_2_O_5_–21.3SiO_2_–*x*CaO–(23.7 − *x*)Na_2_O glasses (mol%; *x* = 0, 12.4, and 23.7 for samples PSi–Na, PSi–NaCa, and PSi–Ca, respectively) were prepared. H_3_PO_4_ (85.0%, solution), SiO_2_ (99.0%), NaH_2_PO_4_ (99.0%) were purchased from Kishida Chemical, Osaka, and CaHPO_4_·2H_2_O (98.0%) was purchased from Fujifilm Wako Pure Chemical, Osaka. The reagents were poured mixed with distilled water (DW) in a Pyrex® beaker to form a slurry. The slurry was stirred and then dried overnight under an infrared lamp to obtain the batch mixtures. Thereafter, the products were melted in a platinum crucible in an electric furnace at 1200 °C for 30 min under atmospheric conditions, after which they were cast onto a stainless-steel plate and subjected to iron-press quenching to obtain the glass samples. The compositions of the samples were analyzed by energy-dispersive spectrometry (EDX, JED-2300, JEOL) (Table 1). The compositions of the resulting glass samples were comparable to their nominal compositions.

**Table 1:** Nominal and analyzed glass compositions. The analyzed compositions are shown in parentheses with their standard deviations

Glass code	Composition (mol%)			
	P_2_O_5_	SiO_2_	Na_2_O	CaO
PSi–Ca	55.0 (54.0 ± 1.1)	21.3 (19.3 ± 1.6)	—	23.7 (26.7 ± 0.7)
PSi–NaCa	55.0 (54.1 ± 1.2)	21.3 (21.2 ± 2.2)	11.3 (11.2 ± 0.7)	12.4 (13.5 ± 1.0)
PSi–Na	55.0 (55.5 ± 0.9)	21.3 (20.4 ± 1.1)	23.7 (24.1 ± 1.5)	—

### 2.3 Spectroscopic analysis of the glasses

Raman spectroscopy (NRS-3300, JEOL, Tokyo) was performed using an Nd:YAG laser to examine the chemical bonding in the glass samples.

MAS-NMR (HNM-ECA A600II, JEOL, Tokyo) analysis was performed to examine the structure around P and Si atoms in the glass samples. ^31^P MAS-NMR was performed using a 3.2 mm probe with a Larmor frequency of 242.95 MHz, spinning at 20 kHz, under the conditions of a single-pulse experiment with a 1.1 μs width, 5.0 s recycle delay, and cumulated number of 256. Ammonium dihydrogen phosphate (NH_4_H_2_PO_4_, 99%; Kishida Chemical, Osaka) was used as a reference at 1 ppm. ^29^Si MAS-NMR was performed using an 8.0 mm probe with a Larmor frequency of 119.24 MHz, spinning at 6 kHz, a 5.0 μs pulse width, and a 120.0 s recycle delay. The accumulation was performed 120–360 times depending on the signal-to-noise ratio of the peak. The chemical shift was adjusted to 1.534 ppm for 4-dimethyl-4-silacyclopentane-1-sulfonic acid sodium salt (C_6_H_13_NaO_3_SSi). For the ^29^Si MAS-NMR measurement, glass samples containing 0.1 wt% MnCO_3_ were prepared and used to shorten the relaxation time.

### 2.4 Number of ions dissolved in a tris-HCl buffer solution

A tris-HCl buffer solution (TBS) at pH 7.40 was prepared by dissolving 6.118 g of tris-hydroxymethylaminomethane [NH_2_C(CH_2_OH)_3_, Kishida Chemical, Osaka] in 1 L of DW at 37 °C and adding 1 M HCl (Kishida Chemical, Osaka). Each glass sample was crushed using an alumina mortar and sieved to particle sizes of 125–250 μm. Next, 20 mg of the glass particles were immersed in 20 mL of TBS and stirred using an incubator shaker (KS4000i, IKA, Osaka) at 37 °C and a speed of 125 rpm for 3 days (*n* = 3). The concentrations of P^5+^, Si^4+^, Ca^2+^, and Na^+^ ions in the TBS after immersion were measured by inductively coupled plasma atomic emission spectrometry (ICP-AES, ICPS-7000, Shimadzu, Kyoto). Although P and Si existed as phosphate and silicate ions in the solution, they were measured as P^5+^ and Si^4+^ ions, respectively, for the convenience of standard reagents.

## 3 Results

### 3.1 Structural analysis with a PSi–Na glass model

The *Q*_P_^*n*^ and Si-coordination number distributions of the simulated model are listed in Table 2. The *Q*_P_^*n*^ distribution was estimated assuming that Si acts as an NWF regardless of the coordination number. The network connectivity (NC),^25^ calculated as the weighted average of the corresponding *Q*_P_^*n*^ distributions, was 2.86, which is close to the experimental value (2.80), indicating that ^[6]^Si in the glass acts as an NWF. Although 8.3% of *Q*_P_^4^ units, which were not observed in the experiment, were observed in the model, the presence of *Q*_P_^4^ units has been reported in several glass systems,^26,27^ and they could be present. However, considering that the amount of *Q*_P_^4^ was small, the simulated and experimental values are comparable. The Si–O coordination number distribution showed 23.8% ^[4]^Si, 9.5% ^[5]^Si, and 70.4% ^[6]^Si. These values are consistent with the experimental results, except for ^[5]^Si, which was not quantitatively evaluated by ^29^Si MAS-NMR.

**Table 2:** *Q*_P_^*n*^ distribution (%), network connectivity (NC), and Si–O coordination number distribution (%) from simulated and experimental results of PSi–Na

	*Q*_P_^*n*^ distribution (%) and NC						Si–O coordination number distribution (%)		
	*Q*_P_^0^	*Q*_P_^1^	*Q*_P_^2^	*Q*_P_^3^	*Q*_P_^4^	NC	^[4]^Si	^[5]^Si	^[6]^Si
Sim.	0.0	0.0	23.1	68.5	8.3	2.86	23.8	9.5	66.7
Exp.	—	—	19.8	80.3	—	2.80	29.4	—	70.9

**Fig. 1:**
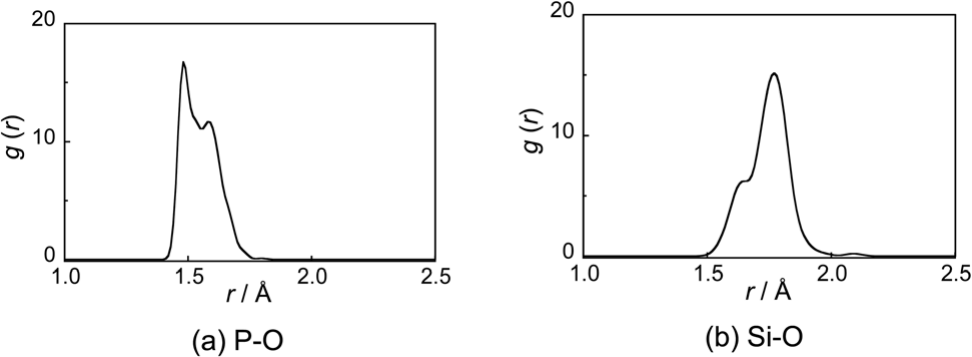
(a) Phosphorus (P)–oxygen (O) and (b) silicon (Si)–O radial distribution functions (RDFs: *g*(*r*)) for the PSi–Na model.

The P–O and Si–O radial distribution functions (RDFs, *g*(*r*)) are shown in Fig. 1. The RDF of P–O shows two peaks at 1.47 and 1.58 Å, which are attributed to the P–NBO and P–BO bonds, respectively, and are consistent with X-ray diffraction results for 50P_2_O_5_–50Na_2_O (mol%) glass.^28^ The RDF of Si–O shows two peaks at 1.64 and 1.77 Å, respectively, which are attributed to the ^[4]^Si–O and ^[6]^Si–O bonds, respectively, and are consistent with X-ray absorption fine structure (XAFS) analysis results for R_2_O–SiO_2_–P_2_O_5_ (R = Li, Na, and K) glass.^9^

**Fig. 2:**
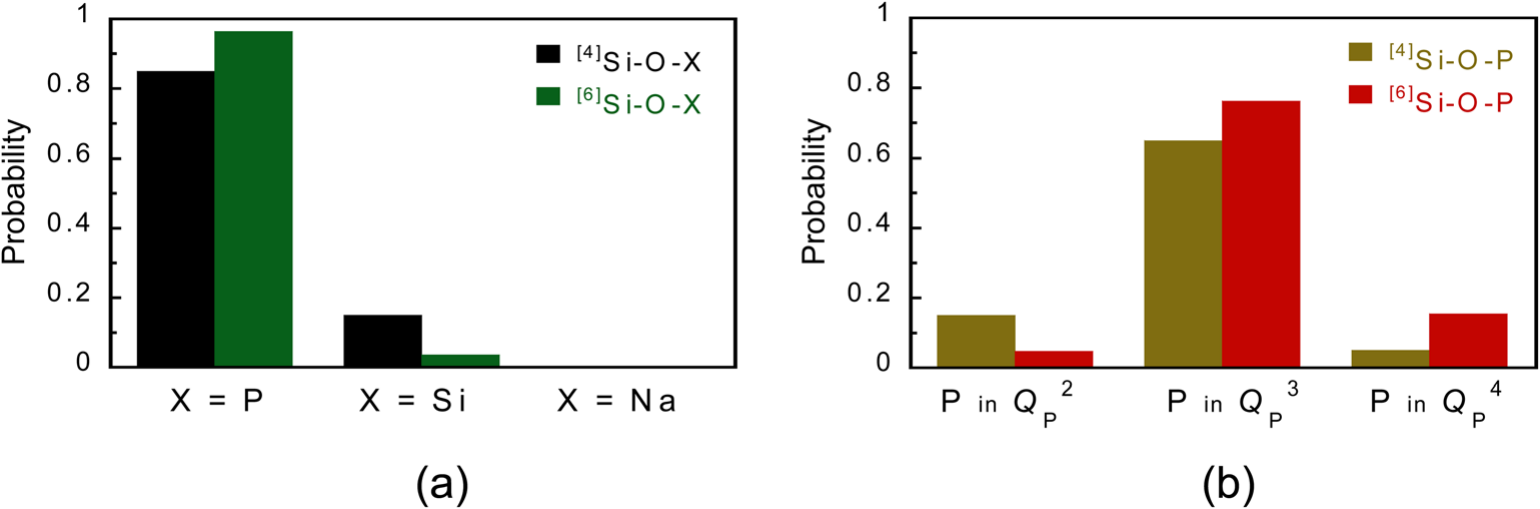
Bridging-type distribution probability of *Q*_P_^*n*^ species estimated from a PSi–Na glass model. (a) Si–O–X (X = P, Si, and Na) and (b) Si–O–P bonds.

The fractions of ^[4]^Si–O–X and ^[6]^Si–O–X (X = P, Si, and Na) bonds are shown in Fig. 2(a). The fractions of ^[4]^Si–O–P and ^[4]^Si–O–Si bonds were 0.85 and 0.15, respectively, which are consistent with the fractions of P and Si in the glass (P : Si = (55.0 × 2) : 21.3), indicating that the phosphate and silicate groups are randomly coordinated to ^[4]^Si. On the other hand, the fractions of ^[6]^Si–O–P and ^[6]^Si–O–Si bonds were 0.96 and 0.04, respectively, indicating that the phosphate group is preferentially coordinated to ^[6]^Si. Fig. 2(b) shows the results divided by the *Q*_P_^*n*^ units bound to Si in Fig. 2(a). For the ^[4]^Si–O–P bond, *Q*_P_^2^ : *Q*_P_^3^ : *Q*_P_^4^ = 17.6 : 76.5 : 5.9, which is consistent with the ratios estimated from the *Q*_P_^*n*^ distribution in the model (Table 2). On the other hand, for the ^[6]^Si–O–P bond, *Q*_P_^2^ : *Q*_P_^3^ : *Q*_P_^4^ = 4.9 : 79.0 : 16.0, with *Q*_P_^3^ showing higher values, indicating that *Q*_P_^3^ units are preferentially coordinated to ^[6]^Si.

**Fig. 3:**
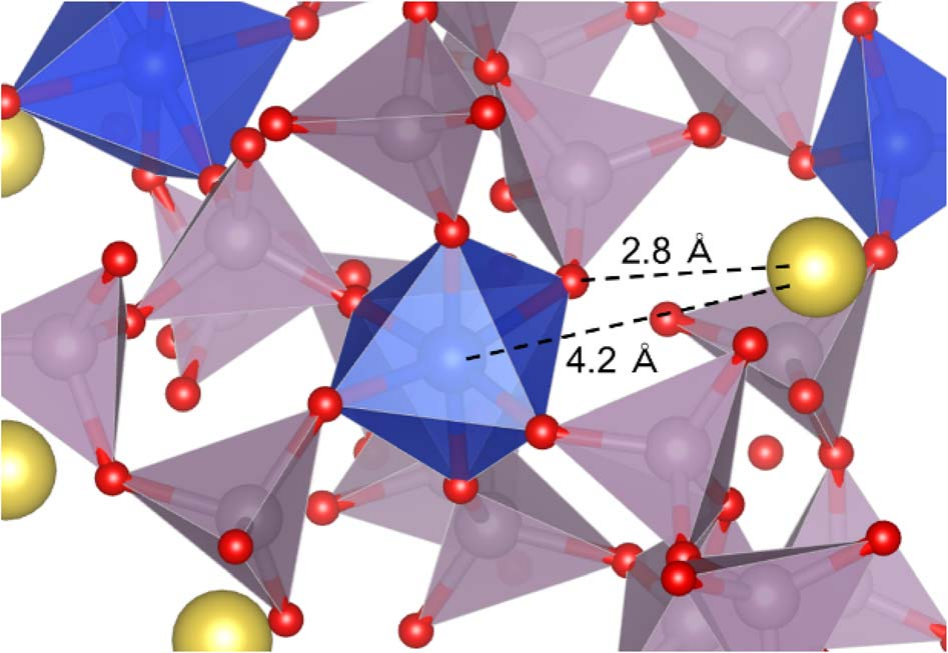
Coordination environment around ^[6]^Si. The numerical values indicate interatomic distances. (Cluster views are provided for a better understanding.) Color legend: P (purple), Si (ivory), Na (yellow), and O (red).

Fig. 3 shows an example of a coordination model around the SiO_6_ octahedron. The Na^+^ is located 4.2 Å away from ^[6]^Si and interacts electrostatically with BO in the ^[6]^Si–O–P bond at an interatomic distance of 2.8 Å.

**Fig. 4:**
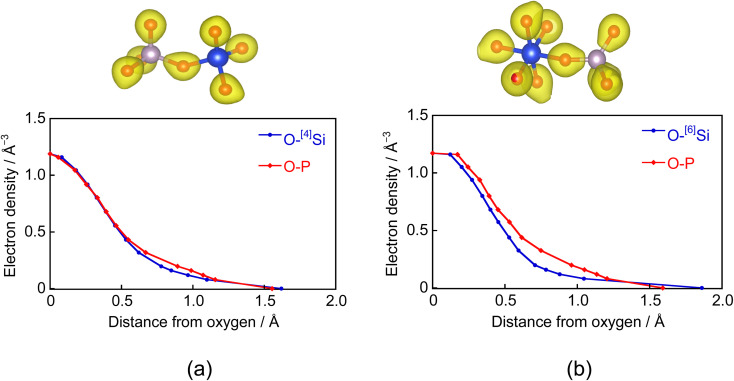
Electron densities between O–P and O–Si atoms in (a) ^[4]^Si–O–P and (b) ^[6]^Si–O–P bonds. (Top): Schematics of the isosurface of the total electron density; (bottom): electron density profiles as a function of distance from oxygen.

Fig. 4 shows the electron densities between the O–Si and O–P atoms in the Si–O–P bond. The horizontal axis represents the distance from BO in the bond direction. Electrons between the O–^[4]^Si atoms in the ^[4]^Si–O–P bond are strongly attracted toward O. However, since the electronegativities of Si and P are close, their distributions are similar. A similar trend was observed between the O–^[6]^Si atoms forming ^[6]^Si–O–P bonds. However, the distribution differs from that between O–P atoms, showing a strong distribution toward the O side. These results indicate that the ^[6]^Si–O bond is more ionic than the ^[4]^Si–O bond. To confirm this, we evaluated the Born effective charges in SiO_2_ crystals *via* first-principles force calculations under an electric field,^29^ similar to our previous study.^30^ Born effective charges for ^[4]^Si and O in alpha-quartz SiO_2_ are 3.46 and −1.73, and those for ^[6]^Si and O in stishovite SiO_2_ are 4.04 and −2.02.

### 3.2 Spectroscopic analysis of the glass structures

**Fig. 5:**
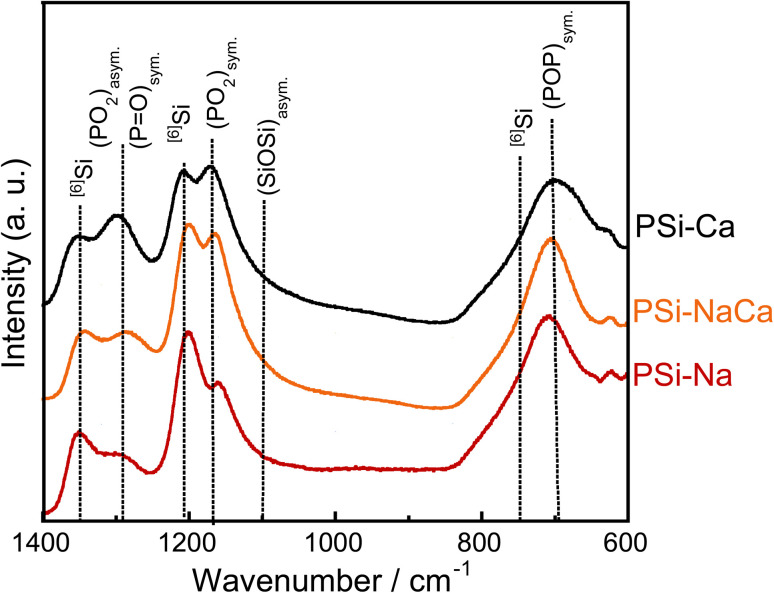
Laser Raman spectra of the glass samples.

Fig. 5 shows the Raman spectra of the glass samples. They show peaks corresponding to P–O–P symmetric vibration around 700 cm^−1^ ((POP)_sym_.),^31,32^^[4]^Si–O–^[4]^Si asymmetric vibration around 1100 cm^−1^ ((SiOSi)_asym._),^33^^[4]^Si–O–^[4]^Si symmetric vibration around 1170 cm^−1^, O–P–O symmetric vibrations of NBO ((PO_2_)_sym._),^31,32^ O–P–O asymmetric vibrations with NBO ((PO_2_)_asym._),^34^ and P=O symmetric vibrations with NBO ((P=O)_sym._)^31,32^ around 1280 cm^−1^. Peaks related to ^[6]^Si are observed around 740, 1200, and 1350 cm^−1^.^35,36^ The peaks originating from NBO vibrations ((PO_2_)_sym._, (PO_2_)_asym._, and (P=O)_sym._) blue-shifted with the addition of CaO, attributed to the weakening of the P–NBO interaction due to the replacement of Na^+^ ion coordinating to NBO with Ca^2+^ ion, which has a higher electronegativity. The peaks related to ^[6]^Si (1200 and 1350 cm^−1^) red-shifted with the addition of Na_2_O.

**Fig. 6:**
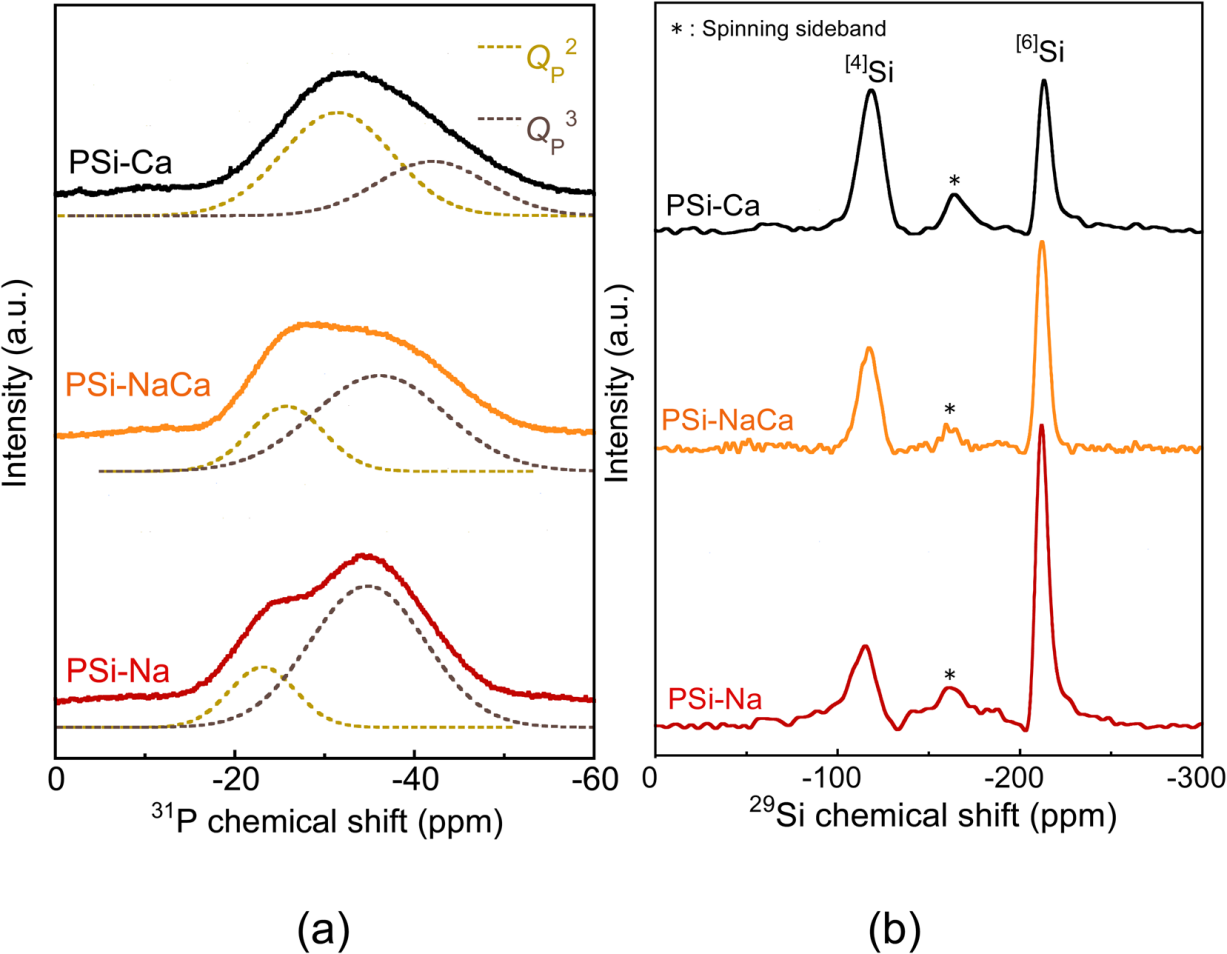
(a) ^31^P and (b) ^29^Si MAS-NMR spectra of the glass samples.

Fig. 6 shows the ^31^P and ^29^Si MAS-NMR spectra of the glass samples. The ^31^P MAS-NMR spectra, which originate from the *Q*_P_^2^ and *Q*_P_^3^ units, were well fitted by two Gaussian functions. The ^29^Si MAS-NMR also showed peaks originating from ^[4]^Si at approximately −120 ppm and ^[6]^Si at approximately −210 ppm. The peak near −160 ppm is ascribed to five-fold-coordinated Si,^37–39^ and it was excluded from the quantitative evaluation since it overlaps with the spinning sideband. Table 3 lists the percentages of the structures estimated from the peaks. The *Q*_P_^3^ unit and ^[6]^Si increased with an increase in the amount of Na_2_O.

**Table 3:** *Q*_P_^*n*^ and Si–O coordination number distributions (%) estimated from the ^31^P and ^29^Si MAS-NMR spectra

Glass code	*Q*_P_^*n*^ distribution (%)		Si–O coordination number distribution (%)	
	*Q*_P_^2^	*Q*_P_^3^	^[4]^Si	^[6]^Si
PSi–Ca	65.4	34.6	62.1	37.9
PSi–NaCa	27.6	72.4	43.7	56.3
PSi–Na	19.7	80.3	29.1	70.9

### 3.3 Dissolution of the glasses in a TBS

**Fig. 7:**
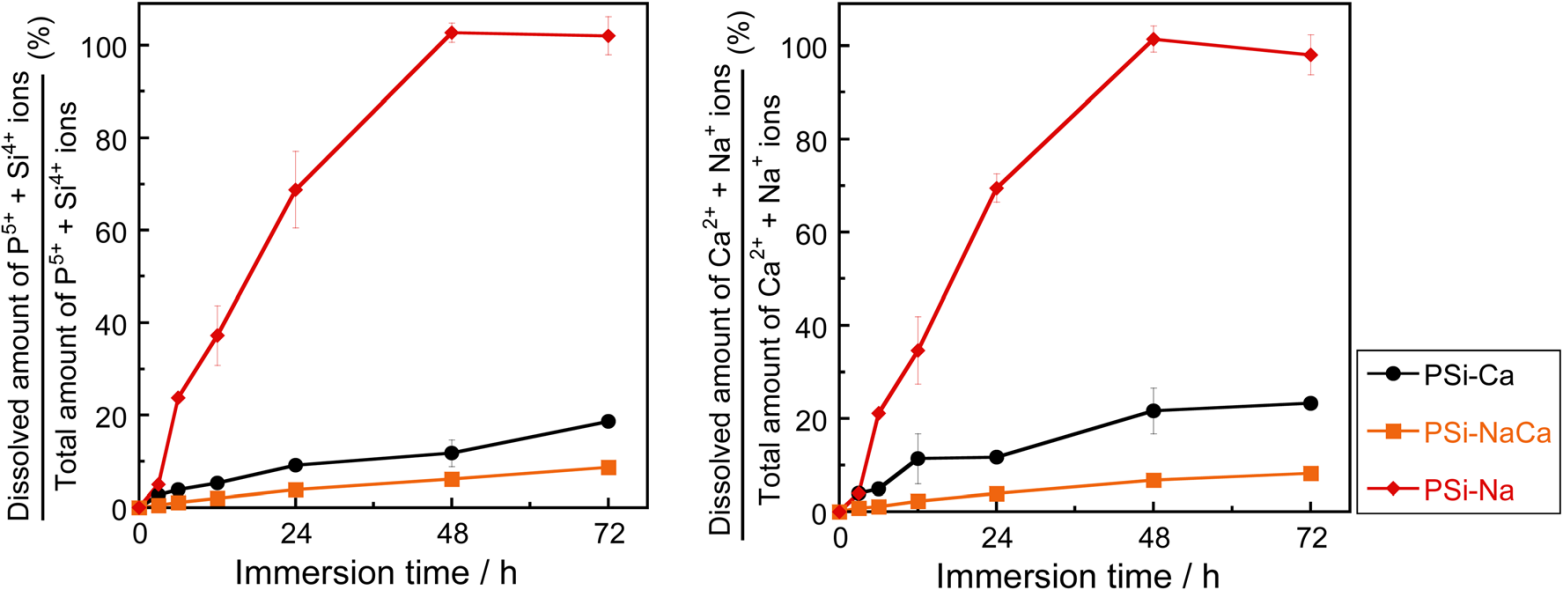
Percentages of ions released in TBS relative to the total amount in the glass samples. Error bar shows the standard deviation.

Fig. 7 shows the percentage of ions dissolved in TBS, which is the ratio of the dissolved ions from the glass to the ion amount in the glass before immersion. The dissolution of the NWF components (P^5+^ and Si^4+^) and the NWM components (Na^+^ and Ca^2+^) increased almost monotonically with time for all glasses. The changes were larger for PSi–Na, PSi–Ca, and PSi–NaCa, in that order. In all samples, the ionic dissolution behavior of the NWF and NWM components were similar and judged to be congruent: almost all of PSi–Na was dissolved after 48 h of immersion. Uo *et al.* reported that binary P_2_O_5_–(100 − *a*)Na_2_O (mol%, *a* = 50–80) glass samples dissolved rapidly within 5 h of immersion in DW; thus, the dissolution of the glass in this study was considerably controlled.^1^ Furthermore, the solubility of PSi–NaCa is suppressed more effectively than that of PSi–Ca, whereas that of P_2_O_5_–CaO glasses without SiO_2_ (ref. 3 and 23) is suppressed with an increase in the CaO content.

## 4 Discussion

### 4.1 Characteristic structures of glass samples containing six-fold-coordinated silicon

^31^P and ^29^Si MAS-NMR analyses revealed increases in the formation of *Q*_P_^3^ units and ^[6]^Si with the addition of Na_2_O. Analysis of the PSi–Na glass model also suggested that ^[6]^Si acts almost exclusively as NWF. These results may indicate that the increase in BO due to the six-fold coordination of Si significantly influences the formation of *Q*_P_^3^ units.

The ^[6]^Si in the glass model preferentially coordinates to the *Q*_P_^3^ unit, and the sharpness of ^[6]^Si peaks in the ^29^Si MAS-NMR spectra could be attributed to this coordination environment, which is consistent with the experimental structural analysis reported by Ren *et al.*^37^ As shown by the RDF of the Si–O bond (Fig. 1(b)), the average of the ^[6]^Si–O interatomic distance is larger than that of the ^[4]^Si–O interatomic distance. Since the electronegativity of P is higher than that of Si, the Si–O interatomic distance would likely be elongated in an environment surrounded by phosphate groups, which favors the formation of ^[6]^Si.

As shown in Fig. 4, the BO in the P–O–^[6]^Si bond generates strong electrostatic interaction with Na^+^ ions due to the high electron density around the bond. In the case of the Na_2_O-containing glass samples, the redshifts of the Raman peaks related to ^[6]^Si (1200 and 1350 cm^−1^) are most likely due to the influence of NWM ions present near the SiO_6_ octahedron.

Considering the Na^+^ ions around ^[6]^Si (within 4.5 Å), as shown in an example in Fig. 3, only one Na^+^ ion was identified in this model. Miyabe *et al.*^40^ proposed a structure with two Na^+^ ions within 3.5–3.8 Å around ^[6]^Si as charge compensation based on molecular orbital cluster simulation. However, their simulation was based on the assumption that the phosphate groups coordinating to ^[6]^Si are terminated with protons without considering the atomic interactions in the range of medium to long distances, which is different in thus study. Zeng *et al.*^36,41^ reported that the *Q*_P_^*n*^ distribution in glasses containing ^[6]^Si strongly depends on the number of NWM ions, and the fraction of ^[6]^Si changes after aging slightly below their glass transition temperature (*T*_g_). Thus, they rejected the structure of SiO_6_ octahedra with two Na^+^ ions as charge compensation. ^[6]^Si forms in binary P_2_O_5_–SiO_2_ glasses containing no NWM ions.^42,43^ Based on these results, we conclude that, although Na^+^ ions have a strong tendency to coordinate around BO in ^[6]^Si–O–P bonds *via* electrostatic interactions, its number is not limited to two.

### 4.2 Relationship between glass structure and the ionic dissolution behavior

As shown in Fig. 7, the solubility of the samples decreased in the order of PSi–Na, PSi–Ca, and PSi–NaCa. In PSi–Na, all Ca^2+^ ions in PSi–Ca and PSi–NaCa were replaced by Na^+^ ions with lower field strength. Thus, the PSi–Na structure is more open than those of PSi–Ca and PSi–NaCa, resulting in the higher solubility of PSi–Na. The solubility of PSi–NaCa was reduced compared to that of PSi–Ca. Ahmed *et al.*^12^ reported that the solubility of P_2_O_5_–Na_2_O–CaO glasses (45 ≤ P_2_O_5_ ≤ 60 (mol%)) increases with increasing Na_2_O content. The coordination state of Si could influence the difference in the dissolution behavior of the glass samples herein from that reported in the previous study.

In the PSi–Na glass model, ^[6]^Si coordinates preferentially to the *Q*_P_^3^ unit. Wazer *et al.*^44^ reported that the *Q*_P_^3^ unit is easily hydrolyzed by H_2_O since the electron distribution around P is localized between P–NBO bonds. In this study, not only the P–O–P bond but also P–O–^[4]^Si and P–O–^[6]^Si bonds are present in the glass samples, and the electron density distribution (Fig. 4) shows that the ^[6]^Si–O bond is highly ionic. Since the electron distribution of P in the ^[6]^Si-coordinated *Q*_P_^3^ unit would be more delocalized compared to that in the P- or ^[4]^Si-coordinated *Q*_P_^3^ units, hydrolysis is reduced. As described in section 4.1, the presence of Na^+^ ions facilitates the ^[6]^Si formation; in PSi–NaCa, the incorporation of Na^+^ ions is considered to increase the ^[6]^Si content, resulting in the controlled solubility.

## 5 Conclusions

We investigated the structure of P_2_O_5_–SiO_2_–Na_2_O–CaO glass *via* theoretical simulation and spectroscopy. We found that ^[6]^Si contributes to the formation of the glass network, producing *Q*_P_^3^ units with a stable electronic configuration, and ^[6]^Si–O bonds are more ionic than ^[4]^Si–O and P–O bonds. *Q*_P_^3^ units are preferentially coordinated to ^[6]^Si, and Na^+^ ions easily coordinate around ^[6]^Si–O–P bonds by interacting with O in the ^[6]^Si–O–P bond. These results show that incorporating ^[6]^Si into P_2_O_5_–SiO_2_–Na_2_O–CaO can alter the electronic state of the phosphate groups and the coordination state of the NWM ions. The solubility of the glass samples in a TBS varied nonlinearly with the Na_2_O content, indicating that the formation of ^[6]^Si could suppress the hydrolysis of the *Q*_P_^3^ units and Na^+^ ion diffusion. Controlling phosphate glass structure using ^[6]^Si is, therefore, an effective technique for tuning the chemical dissolution of the glass. In P_2_O_5_–SiO_2_–Na_2_O–CaO glass, the ^[6]^Si content and *Q*_P_^*n*^ distribution can be controlled by balancing Na_2_O and CaO contents.

## Author contributions

All authors contributed to the writing of this manuscript and have approved its final version. K. T.: data curation, formal analysis, visualization, and writing – original draft; T. T.: methodology and writing – review and editing; T. K.: conceptualization, methodology, and writing – review and editing.

## Conflicts of interest

The authors declare no competing interests.

## Supplementary Material
